# Cardioprotective effect of *Platycodon grandiflorum* in patients with early breast cancer receiving anthracycline-based chemotherapy: study protocol for a randomized controlled trial

**DOI:** 10.1186/s13063-017-2140-z

**Published:** 2017-08-22

**Authors:** Wei Hao, Sheng Liu, Yuenong Qin, Chenping Sun, Liying Chen, Chunyu Wu, Yijia Bao

**Affiliations:** Department of Breast Surgery (Integrated Traditional and Western Medicine), Longhua Hospital affiliated to Shanghai University of Traditional Chinese Medicine, 725 South Wanping Road, Xuhui District, Shanghai, 200032 China

**Keywords:** Anthracyclines, Cardiotoxicity, *Platycodon grandiflorum*, Protocol, Herbal medicine

## Abstract

**Background:**

Anthracyclines, alone or in combination with other drugs, are among the most effective chemotherapeutic agents to treat breast cancer both in the adjuvant and neoadjuvant setting. Unfortunately, anthracycline-associated dose-dependent cardiotoxicity is a limiting factor in clinical use. Extensive efforts have been devoted to identifying strategies to prevent anthracycline-induced cardiotoxicity. However, most cardioprotective agents have shown little effect in clinical trials. Herbal medicines are pure, natural substances that have been used for centuries in many countries, including China. This trial aims to evaluate the cardioprotective effects and safety of *Platycodon grandiflorum* granules compared to placebo granules in patients with early breast cancer receiving anthracycline-based chemotherapy.

**Method/design:**

This study is a single-center, double-blinded, randomized, placebo-controlled, parallel-group trial. A total of 120 patients will be randomly allocated in a 1:1 ratio to receive either *P. grandiflorum* granules or placebo granules twice daily for 12 weeks. The primary outcome is heart failure (either clinical or subclinical). The secondary outcomes include all-cause mortality, cardiac death, electrocardiogram (ECG) findings, left ventricular diastolic function, longitudinal systolic strain and velocities measured by tissue Doppler imaging, cardiac biomarkers, such as troponin I (TnI), brain natriuretic peptide (BNP), and creatine kinase isoenzymes (CK-MB). Assessments will be performed at baseline (before randomization) and 3, 6, 9, 12, 16, and 20 weeks after randomization.

**Discussion:**

This will be the first clinical trial to evaluate the cardioprotective effects and safety of *P. grandiflorum* in patients with early breast cancer receiving anthracycline-based chemotherapy. We are also performing this trial to assess the feasibility of a larger-scale clinical trial in the future.

**Trail registration:**

Chinese Clinical Trial Registry, ChiCTR-IPR-16009256. ﻿Registered on 23 September 2016.

**Electronic supplementary material:**

The online version of this article (doi:10.1186/s13063-017-2140-z) contains supplementary material, which is available to authorized users.

## Background

Anthracycline (ANT) compounds (e.g., doxorubicin (DOX), epirubicin (EPI), and daunorubicin (DNR)) are some of the most effective antineoplastic drugs in the treatment of both hematological malignancies and solid tumors [1]. Moreover, anthracycline-based treatments are first-line chemotherapy agents to treat early breast cancer [2]. Unfortunately, the clinical use of anthracyclines is limited in some patients by the risk of severe cardiotoxicity, especially in patients receiving high cumulative drug doses [3].

Cardiac complications after DNR exposure were first reported in 1967 [4]. Since then, accumulating evidence has deemed cardiotoxicity a side effect common to all ANTs. Despite almost 50 years of efforts to prevent anthracycline-induced cardiotoxicity, there still remains considerable controversy regarding the best way to do so. Various potential cardioprotective agents, including dexrazoxane, coenzyme Q10, vitamin E, angiotensin converting enzyme (ACE) inhibitors, statins, and beta blockers, have been well-studied to determine their cardioprotective effects [5–10]. A Cochrane systematic review concluded that the only agent to have a cardioprotective effect was dexrazoxane (risk ratio (RR) 0.18, 95% confidence interval (CI) 0.10–0.32, *P* < 0.00001) [11]. But dexrazoxane is only approved by the US Food and Drug Administration (FDA) for women with metastatic breast cancer who havereceived at least 300 mg/m^2^ doxorubicin and need additional doxorubicin to maintain tumor control. Additionally, dexrazoxane is not widely used even for this indication due to cost, especially in developing countries [12]. Moreover, dexrazoxane is not without its own side effects, including the potential increased risk of secondary malignancy [13].

In the absence of effective, affordable therapies, some physicians in China turn to alternative or complementary treatments to prevent anthracycline-induced cardiotoxicity. Chinese herbal medicine (CHM) is an essential part of traditional Chinese medicine (TCM), and has been widely used in China for centuries. According to TCM theory, patients with anthracycline-induced cardiotoxicity are diagnosed as “Xiong bi.” “Qi deficiency” is the major syndrome. *Platycodon grandiflorum* (*jiegeng*) is an herb that has been used in TCM for thousands of years to treat cardiovascular disease. In TCM theory, *P. grandiflorum* can nourish Qi and relieve symptoms, such as palpitations, shortness of breath, and chest pain.

The molecular basis for cardiotoxicity after ANT exposure remains unclear. The most widely accepted hypothesis for anthracycline-induced cardiotoxicity is the generation of excess reactive oxygen species (ROS) and redox cycling [14]. However, a series of in vitro and in vivo studies investigating treatment with antioxidants or iron chelation agents failed to prevent cardiac toxicity caused by doxorubicin [15, 16]. Some studies found that extract from *P. grandiflorum* has stronger antioxidant effects than conventional antioxidants. *P. grandiflorum* can reduce formation of ROS and lipid peroxidation [17, 18]. Additional studies found that too much or too little nitric oxide (NO) might damage myocardial cells, and *P. grandiflorum* can maintain the concentration of NO in myocardial cells [19–22].

Therefore, *P. grandiflorum* may have the potential to prevent anthracycline-induced cardiotoxicity. Compared with dexrazoxane, *P. grandiflorum* has the advantage of being more affordable. However, there have been no high-quality clinical trials that evaluate the safety and efficacy of *P. grandiflorum*. This study aims to evaluate the cardioprotective effects and safety of *P. grandiflorum* compared to placebo in patients with early breast cancer receiving anthracycline-based chemotherapy. This trial was also performed to assess the feasibility of a large-scale clinical trial that may provide alternative, evidence-based prevention of anthracycline-induced cardiotoxicity.

## Methods/design

### Study objective

The primary objective of this study is to ascertain the potential cardioprotective effects and safety of *P. grandiflorum* compared to placebo in patients with early breast cancer receiving anthracycline-based chemotherapy. The secondary objective is to determine whether there are other possible effects of *P. grandiflorum* on protecting against other, non-cardiac anthracycline toxicities.

### Hypothesis

We hypothesize that: (1) after 12 weeks, fewer patients taking *P. grandiflorum* will develop cardiac damage compared with patients receiving placebo, (2) *P. grandiflorum* will be safe at the dosage used in this trial, and (3) *P. grandiflorum* will have similar effects to placebo on non-cardiac anthracycline toxicities.

### Study design

**Fig. 1:**
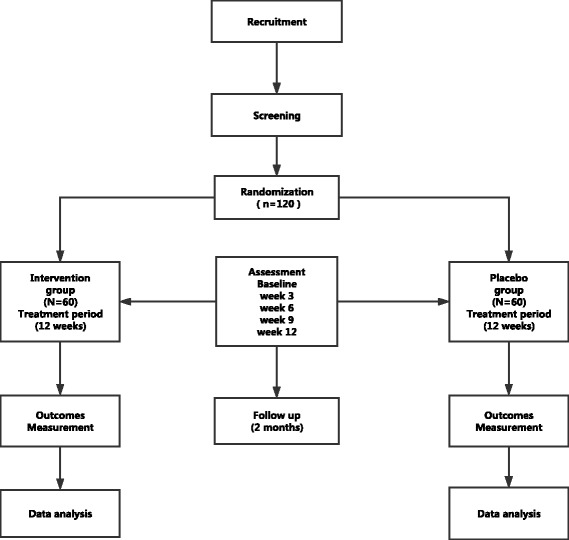
Flow chart of the study

This is a single-center, double-blinded, randomized, placebo-controlled, parallel-group trial. Figure 1 depicts a flow chart of the study. A Standard Protocol Items: Recommendations for Interventional Trials (SPIRIT) checklist for this protocol is also provided (see Additional file 1).

### Recruitment

The study will be performed in the Breast Surgery Unit (Integrated Traditional Chinese and Western Medicine) of Longhua Hospital affiliated with Shanghai University of Traditional Chinese Medicine. The study will identify patients diagnosed with early breast cancer at our center who are receiving anthracycline-based chemotherapy. Patients receiving either adjuvant or neoadjuvant chemotherapy will be recruited. Participants who initially present to our hospital through both inpatient and outpatient units will be screened. Eligible patients who agree to participate will be asked to sign an informed consent form. To achieve adequate participant enrollment, study drug will be provided to participants at no cost. No other financial incentives will be provided to either treating physicians or patients.

### Eligibility criteria

#### Inclusion criteria

Patients will be eligible for inclusion if they meet all of the following criteria:

1. Histological or cytological proven breast cancer
2. Receiving anthracycline-based chemotherapy
3. Karnofsky Performance Status (KPS) ≥ 60
4. Life expectancy ≥ 6 months
5. Aged 20–70 years
6. Willing and able to give informed consent

#### Exclusion criteria

Patients will be excluded if any of the following occurs:

1. History of myocardial injury, coronary artery disease (CAD), myocarditis, or functional cardiac insufficiency
2. Previous anthracycline-based chemotherapy or radiotherapy
3. Previous treatment with any investigational drug within 3 months prior to receiving the first dose of *P. grandiflorum* (or placebo)
4. Metastatic disease
5. Pregnancy
6. Lack of compliance
7. Allergic to investigational drugs
8. Participation in other clinical trial(s) within 3 months prior to this trial

### Randomization, allocation concealment, and blinding

To minimize selection bias, all eligible patients will be randomized in a 1:1 ratio to the treatment group or control group. We will use IBM SPSS software version 19 (IBM, Armonk, NY, USA) for group allocation according to a randomization list. Randomization will be performed by a professional, independent statistician who is not involved in the recruitment process of the study. The randomization codes will be concealed in sequentially prepared, coded, individual, opaque envelopes prepared by research assistants who are not involved in the recruitment process. The envelopes will be kept secure in the Good Clinical Practice (GCP) Centre of Longhua Hospital and will not be opened until after statistical analysis or in the case of a medical emergency. The study participants, study investigators, other research team members, pharmacists, nurses, and technicians will remain blinded to the allocation of study medication versus placebo.

### Study drug

CHM extract granules have been used in China for many years. In this study, the *P. grandiflorum* and placebo granules used were both manufactured by Jiangyin Tianjiang Pharmaceutical Co., Ltd (Jiangyin, China), a manufacturer of CHM extract granules that has been certified for Good Manufacturing Practice. *P. grandiflorum* granules have been approved by the China Food and Drug Administration (CFDA). Quality control was conducted by the company. *P. grandiflorum* granules contain a single herb, *P. grandiflorum*. A raw *P. grandiflorum* herb weighing 3 g was cooked in boiling water. The residual was then filtered, concentrated, spray-dried, and mixed with starch and sucralose. Finally, the *P. grandiflorum* granules were packaged in small pouches weighing 2 g each. The placebo granules are composed of 10% *P. grandiflorum* (a sub-therapeutic dose) and 90% starch, giving the placebo an identical appearance, smell, taste, and packaging as the *P. grandiflorum* granules.

### Intervention

Enrolled patients will be randomly allocated to either the treatment or control group. Patients in the treatment group will receive anthracycline-based chemotherapy with *P. grandiflorum* granules. Patients in the control group will receive anthracycline-based chemotherapy with placebo granules. All patients, regardless of which group they are randomized to, will be instructed to dissolve one package of granules in 150 mL hot water twice daily for 12 consecutive weeks. Participants will be withdrawn if they take any other CHM or Western medication to treat heart disease during the 12-week intervention period or if they are not willing to take the study drug. Treatment for other conditions, such as hypertension, diabetes mellitus, and other chronic diseases, will be permitted during the intervention. The name, duration, and dosage of any other drugs taken during the 12-week intervention period will be recorded in each patient’s case report form (CRF). In order to assess adherence to taking the intervention medication, each participant will be asked to undergo laboratory tests at each follow-up appointment. Patients will also be asked to return any unused drug granules. Participants who return more than 10% of the total allotted granules will be defined as nonadherent. Patient-reported reasons for nonadherence will be recorded on the CRF.

### Outcomes

#### Primary outcome

The primary outcome is heart failure (either clinical or subclinical). In this study, New York Heart Association (NYHA) Functional Classification will be used to evaluate the extent of clinical heart failure. Left ventricular ejection fraction (LVEF) less than 50% or a drop of more than 15% from baseline is defined as subclinical heart failure in this study. Ejection fraction is measured using multigated acquisition (MUGA) scan in all participants. MUGA scans will be performed by the same physician; this physician is not involved in recruitment of the study.

#### Secondary outcomes

Secondary outcomes include all-cause mortality, cardiac death, electrocardiogram (ECG) findings, left ventricular diastolic function, longitudinal systolic strain and velocities measured by tissue Doppler imaging, cardiac biomarkers, such as troponin I (TnI), brain natriuretic peptide (BNP), and creatine kinase isoenzymes (CK-MB) [23].

#### Safety outcomes

Safety outcomes include both clinical and subclinical measures. Routine physical examination will be performed on all participants during the study period; any unexpected symptoms or signs will be recorded as clinical measures. Routine complete blood count, blood chemical analysis, urinalysis, fecal examination, liver function tests, and renal function tests will be performed as subclinical measures to assess safety for all participants.

### Data collection and management

**Fig. 2:**
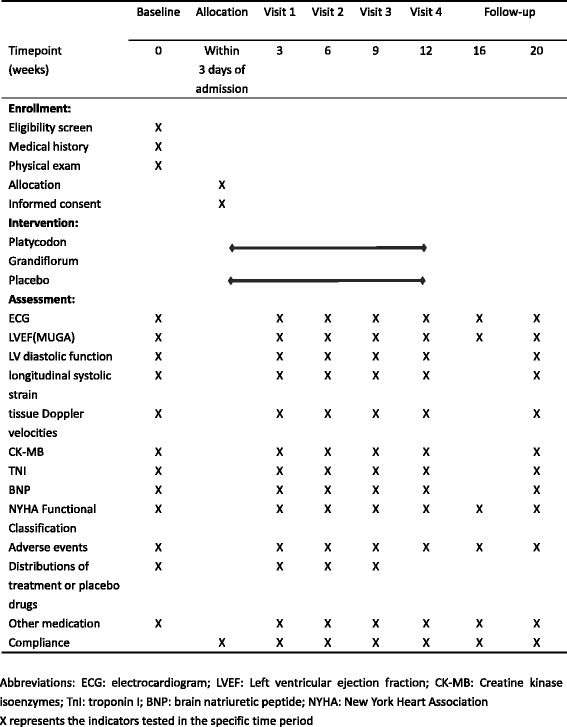
Schedule of enrollment, interventions and assessments (using the SPIRIT 2013 statement [26]). ECG electrocardiogram, LVEF left ventricular ejection fraction, MUGA multigated acquisition, LV left ventricle, CK-MB creatine kinase isoenzymes, TNI troponin I, BNP brain natriuretic peptide, NYHA New York Heart Association

CRFs will be used to collect data. To promote data quality and accuracy, one trained investigator will complete the CRFs and a second investigator will independently review all CRFs. Once data collection is complete, all data will be entered in duplicate into a computer database using EpiData 3.1 software (The EpiData Association, Denmark, Europe), by two individual data managers working independently through separate authorized identification codes and passwords. Figure 2 summarizes the study schedule of enrollment, interventions, and assessments according to the SPIRIT 2013 statement. Regular backups and off-site storage will be performed. The original CRFs and any other paper records will be kept on file at the clinical study center for 5 years after completion of the study.

### Data and safety monitoring

Both adverse events (AEs) and serious adverse events (SAEs) will be assessed in this study. AEs are defined as unintended or unfavorable clinical symptoms, signs, laboratory results, or diseases that do not necessarily have a causal relationship with the study intervention. AEs in all participants in this clinical trial will be recorded at every visit. Details of all AEs will be recorded, including starting and ending date, grade (using the National Cancer Institute “Common terminology criteria for adverse events (NCI-CTCAE v 4.03)” [24]), and relation to the study medication. SAEs are defined as death, illness requiring hospitalization, events deemed life-threatening, events that result in persistent or significant disability or incapacity, a congenital anomaly or birth defect, or other important medical conditions. The Data and Safety Monitoring Board (DSMB) for this study will comprise one independent statistician, one senior pharmacist, one physician, and one individual with experience supervising a clinical trial. All members of the DSMB are independent from the trial sponsor and have no competing interests. The DSMB will organize a conference before allocation of the study and will review the trial documentation at least once to ensure that the trial is being conducted according to the trial protocol and GCP principles and that the data and SAEs are accurately and appropriately recorded on the CRF. All SAEs will be reported to the DSMB, ethics committees, sponsors, and the CFDA immediately. If a patient experiences a SAE, the DSMB has permission to unblind the case in the event of a medical emergency.

### Sample size calculation

The study aims to detect the cardioprotective effects of *P. grandiflorum* in patients with early breast cancer receiving anthracycline-based chemotherapy. Sample size was calculated on the basis of the primary outcome. Since no randomized controlled trials (RCTs) have been previously performed on the cardioprotective effects of *P. grandiflorum* in patients receiving anthracycline-based chemotherapy, we proposed this study to evaluate the feasibility of a large-scale clinical trial. Anticipating a 20% dropout rate, a total of 120 patients, with 60 patients in each group, will be recruited for the pilot study.

### Statistical analysis

An independent, professional statistician will perform the data analysis for the results and the AEs. Intention-to-treat (ITT) analysis will be carried out in all participants who complete the clinical trial. Missing data will be adjusted for using the last observation carried forward (LOCF) principle to obtain a complete database. Continuous data will be presented as means and standard deviations $\left(\overline{X}\pm SD\right)$, or medians and quartiles. The comparability of the two groups will be assessed using the independent samples *t* test for continuous variables and the chi-square (χ^2^) test or Wilcoxon test, when appropriate, for categorical variables. The cumulative dose of anthracycline to cause cardiotoxicity will be estimated in each group using Kaplan-Meier curves; differences between the curves will be assessed using the log-rank method. The crude and adjusted hazard ratios and 95% confidence intervals will be estimated using a Cox proportional hazards regression model. All analyses will be performed using IBM SPSS software version 19 (IBM, Armonk, NY, USA). A two-tailed *P* value <0.05 was set for statistical significance.

## Discussion

Since *P. grandiflorum* has never been studied for the prevention of cardiotoxicity in patients receiving anthracycline-based chemotherapy, we performed a randomized controlled trial to evaluate the cardioprotective effects and safety of *P. grandiflorum* in patients with early breast cancer receiving anthracycline-based chemotherapy. We also plan to assess the feasibility of a future, larger-scale clinical trial. Despite having no precedent of a clinical trial assessing our intervention, this protocol adheres to strict, high-quality methodology and follows the Consolidated Standards of Reporting Trials (CONSORT) statement for RCTs of herbal medicines [25] and the SPIRIT 2013 statement [26]. Key elements for performing a high-quality RCT have been described above in detail, such as randomization, allocation concealment, blinding methods, sample size, intervention, outcome measures, and statistical analysis. There are some limitations to our study. First, patients with breast cancer have longer overall survival (OS) and progression-free survival (PFS) than patients with other kinds of cancer. Since the follow-up period of this study is relatively short, we may not be able to compare OS and PFS between the two groups. Second, the gold standard for diagnosing anthracycline-induced cardiotoxicity is endomyocardial biopsy (EMB). However, EMB carries risk of serious complications and thus EMB is limited in its clinical application. Surrogate markers of cardiotoxicity may be easier to perform and carry lower risk, but also may result in more false-positive or false-negative results in both groups.

### Trial status

Participant recruitment and randomization began in November 2016. Recruitment is ongoing.

## Additional file

Additional file 1: SPIRIT checklist. (DOC 115 kb)

## Abbreviations

AE: Adverse event
ANT: Anthracycline
BNP: Brain natriuretic peptide
CAD: Coronary artery disease
CFDA: China Food and Drug Administration
CHM: Chinese herbal medicine
CK-MB: Creatine kinase isoenzymes
CONSORT: Consolidated Standards of Reporting Trials
CRF: Case report form
DNR: Daunorubicin
DOX: Doxorubicin
DSMB: Data and Safety Monitoring Board
EMB: Endomyocardial biopsy
ECG: Electrocardiogram
EPI: Epirubicin
GCP: Good Clinical Practice
ITT: Intention-to-treat
KPS: Karnofsky Performance Status
LOCF: Last observation carried forward
LVEF: Left ventricular ejection fraction
MUGA: Multigated acquisition
NO: Nitric oxide
NYHA: New York Heart Association
OS: overall survival
PFS: Progression-free survival
RCT: Randomized controlled trial
ROS: Reactive oxygen species
SAE: Serious adverse event
SPIRIT: Standard Protocol Items, Recommendations for Interventional Trials
TCM: Traditional Chinese medicine
TnI: Troponin I
